# Structural and Functional Responses of Root Endophytic Fungi to Fairy Ring Expansion in Alpine Meadows on the Tibetan Plateau

**DOI:** 10.1002/ece3.74301

**Published:** 2026-09-16

**Authors:** Chengxing Wu, Zhihua Wu, Shiting Cao, Sisong Tu, Yuying Zhang, Na Li, Ming Wang, Huiyuan Ren, Qingbo Gao, Shilong Chen, Rui Xing

**Affiliations:** ^1^ Northwest Institute of Plateau Biology, Chinese Academy of Sciences Xining Qinghai China; ^2^ College of Life Science University of Chinese Academy of Sciences Beijing China; ^3^ Qinghai Nationalities University Xining Qinghai China; ^4^ Qinghai Provincial Key Laboratory of Crop Molecular Breeding Xining Qinghai China

## Abstract

Fairy rings (FRs) are potent ecosystem engineers that reshape soil environments, yet the response of root endophytic fungi (REF) to these perturbations remains less understood. Here, we characterized REF communities in *Kobresia humilis* across three FR types—Type I (injurious *Agrocybe* sp.), Type II (promoting *Agaricus campestris*), and Type III (neutral *Clitocybe* sp.)—on the Tibetan Plateau. Our data suggest that, unlike rhizosphere microbial communities that are often influenced by host recruitment, REF communities may be more strongly shaped by interactions among fungal taxa. Specifically, we observed a consistent decline in the relative abundance of putatively beneficial fungal groups, including Glomeromycetes and Archaeorhizomycetes, within fungal‐active (ON) zones. We propose the term “Symbiotic Decoupling” as a conceptual hypothesis to describe this pattern, whereby FR expansion may be associated with a reduced representation of these taxa in root endophytic fungal communities. This pattern may be consistent with different ecological trade‐offs associated with contrasting FR environments. In promoting Type II rings, elevated nutrient availability (e.g., phosphorus) coincided with a simplified network topology. This pattern is consistent with the hypothesis that increased nutrient availability may reduce the relative importance of certain symbiotic associations, a concept we tentatively refer to as “Resource‐driven Intensification”. Conversely, the T1 network displayed a comparatively denser topology; however, because the inferred networks differed in node and edge numbers, these differences should not be interpreted as direct quantitative evidence of greater connectivity. This pattern may indicate increased coordination among community members under stressful conditions and is consistent with a conceptual framework that we term “Defensive Complexity”. However, the functional significance of this network configuration remains to be experimentally validated. Collectively, these findings suggest that FR expansion may alter the balance between host‐associated filtering processes and fungal community interactions, potentially resulting in community configurations that favor rapid resource acquisition or persistence over the maintenance of previously dominant symbiotic taxa.

## Graphical abstract

This study characterizes root endophytic fungal communities across three fairy ring types on the Tibetan Plateau, revealing that community assembly is driven by intra‐kingdom competition that leads to “Symbiotic Decoupling” and the suppression of native mutualists in fungal‐active zones. We found that communities adopt distinct topological strategies based on the ring type, exhibiting simplified “Resource‐driven Intensification” in nutrient‐rich promoting rings and hyper‐connected “Defensive Complexity” in injurious rings to buffer against stress.

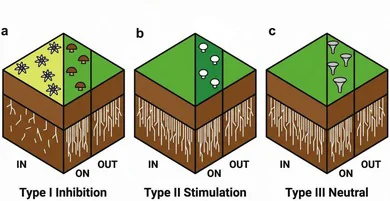

## 1 Introduction

Fairy rings (FRs) are striking circular or arc‐shaped formations created by the dynamic, radial expansion of fungal mycelia in soil. As a prevalent ecological phenomenon, particularly within grassland and woodland ecosystems, FRs serve as significant drivers of spatial heterogeneity (Gregory 1982). Approximately 50 fungal species across genera such as *Agaricus*, *Agrocybe*, *Clitocybe*, *Floccularia*, and *Leucocalocybe* have been reported to form FRs (Marí et al. 2020; Xing et al. 2023). FRs are often categorized into three types based on their potential effects on vegetation: Type I (Injurious): these rings cause extensive damage to vegetation, characterized by a zone of wilting, necrotic, or deceased flora within or on the ring. Type II (Promoting): these are characterized by a conspicuous band of lush, dark‐green vegetation where plant growth is significantly stimulated. Type III (Neutral): these formations exhibit no discernible impact on the surrounding vegetation, manifesting only as a circular arrangement of fungal fruiting bodies (Shantz 1917). These formations offer a natural model to study interactions among plants, soil, and microorganisms. Previous studies have mainly focused on Type II FRs, suggesting that species like *Marasmius oreades* (Fisher 1977) and *Agaricus arvensis* (Edwards 1988) may influence nutrient availability during mycelial growth, potentially affecting plant growth. *Leucocalocybe mongolica* can drive bacterial succession toward plant‐growth‐promoting rhizobacteria (PGPR), such as *Bacillus pumilus* , and even enhance host salt stress resistance through metabolic up‐regulation (Wang, Wang, et al. 2022). In temperate grasslands, *Agaricus gennadii* (Yang et al. 2018) dramatically elevates soil pH and effective phosphorus, indirectly regulating plant distribution by modifying spatial nutrient heterogeneity.

The Tibetan Plateau, often called the “Third Pole” due to its extreme climate and high elevation, supports extensive alpine meadows that are ecologically and socioeconomically important (Qiu 2008). These meadows contribute to regional biodiversity and ecosystem stability and provide a foundation for pastoral livelihoods (Immerzeel et al. 2020). However, these fragile high‐altitude ecosystems are currently facing severe degradation. Addressing this ecological challenge requires synergistic strategies that balance environmental sustainability with economic development through scientific grassland management (Bardgett et al. 2021). In this context, research into FR fungi offers a unique natural model for enhancing soil nutrient availability and enzyme activity, which may facilitate the restoration of degraded lands and improve the productivity of alpine meadows. Research on FRs in alpine meadows has progressed from macro‐ecological assessments to molecular investigations. Early studies suggested that *Floccularia luteovirens* rings can modify soil properties and plant communities (Wang et al. 2005). High‐throughput sequencing later revealed that radial fungal growth may coincide with changes in microbial diversity, potentially linked to shifts in soil physicochemical characteristics (Xing et al. 2018). Subsequent research by Cao et al. (2021) further elucidated that the biological influence of *F. luteovirens* on the soil–plant interface is mediated by specific modifications in glucose and amino acid metabolism (Cao et al. 2021). More recent studies on *Agaricus xanthodermus* indicate that the ecological effects of FRs, on both vegetation and microbial communities, appear context‐dependent (Du et al. 2024).

Soil microbial communities are organized into distinct micro‐niches, with the rhizosphere and the endosphere representing two of the most critical zones for plant‐microbe interactions (Bulgarelli et al. 2012). The rhizosphere, the thin layer of soil influenced by root exudates, is highly dynamic and sensitive to changes in soil physicochemical properties and external biotic factors (Philippot et al. 2013). In contrast, endophytic microorganisms colonize internal root tissues and can interact closely with the host, potentially influencing plant physiological processes (Hardoim et al. 2015). We previously demonstrated that the expansion of FR fungi significantly alters the diversity and composition of soil bacterial and fungal assemblages across different zones (Xing et al. 2018). However, the role of root endophytic microbes in the context of FR‐mediated stress remains a critical knowledge gap. Microbial endophytes (especially the root endophytic fungi, REF) are uniquely distinguished from their rhizosphere counterparts by their specialized ecological functions. REF are thought to contribute to phosphorus acquisition and produce bioactive secondary metabolites that may protect plants against pathogens (Orozco‐Mosqueda et al. 2025) and could play a role in plant stress resilience under nutrient fluctuations induced by FR growth (F. Liu et al. 2021).

Despite progress in characterizing the rhizosphere microbiota, knowledge gaps persist regarding REF. Because REF and FR fungi both belong to the fungal kingdom, they may share similar ecological niches or resource requirements (Graziosi et al. 2025; van der Wal et al. 2013), raising the possibility of competitive interactions. Similarly, FR expansion might influence native mycorrhizal fungi through competition for root colonization or carbon resources (Martin and van der Heijden 2024). Additionally, FR fungal activity may alter local soil chemistry or metabolite profiles, which could affect mycorrhizal associations or nutrient dynamics, although these effects remain to be tested (Zotti et al. 2025). To address these gaps, this study evaluates structural and functional responses of REF communities across three FR types—*Agrocybe* sp. (T1), *Agaricus campestris* (T2), and *Clitocybe* sp. (T3)—on the Tibetan Plateau. Samples were collected from the inner (IN), beneath‐ring (ON), and outer (OUT) zones to provide a framework for understanding how root fungal endophytes may respond to radial mycelial expansion.

## 2 Material and Methods

### 2.1 Study Site Description and Sampling

**FIGURE 1:**
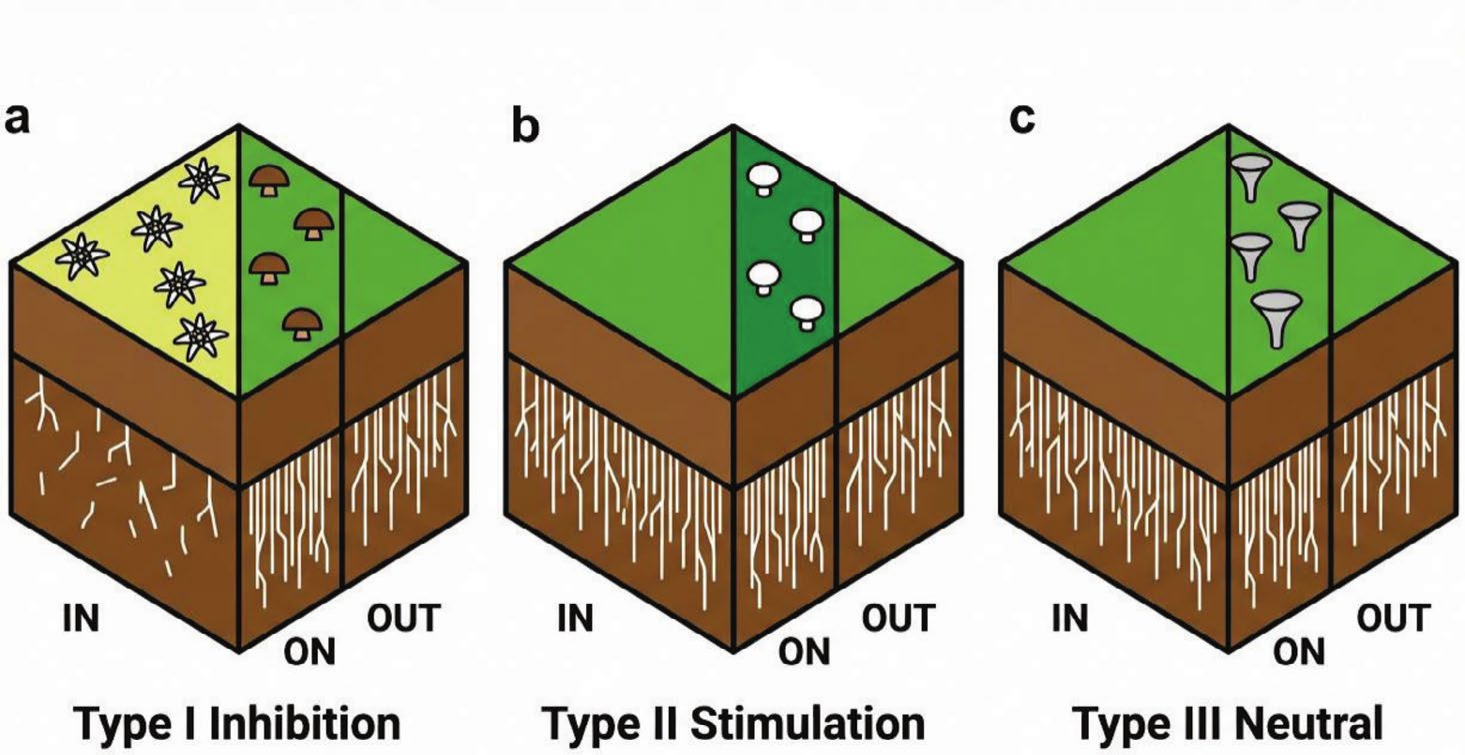
Schematic representation of sampling designs across three types of fairy rings (FRs). (a) Type I (T1): Inhibitory ring characterized by a necrotic zone with reduced plant coverage, formed by *Agrocybe* sp. (b) Type II (T2): Promotional ring exhibiting a lush vegetation belt, formed by *Agaricus campestris*. (c) Type III (T3): Neutral ring identified solely by fruiting bodies without discernible vegetation shifts, formed by *Clitocybe* sp. For each FR type, soil was harvested from three zones: ON (directly beneath the fruiting bodies), IN (20 cm interior to the ON zone), and OUT (20 cm exterior to the ring, serving as the local control).

The field investigation was conducted within the alpine meadow ecosystems on the northern fringes of the Tibetan Plateau (Yeniugou, Qinghai Province). The ecological characteristics of this study area are consistent with our previous research on *Floccularia luteovirens* FRs in the same locale (Xing et al. 2023). Based on the distinct vegetation responses and fungal species involved, three representative FR types were selected for analysis: (i) T1 formed by *Agrocybe* sp., characterized by a deleterious effect where the original dominant community (*Poa annua, Thermopsis lanceolata*, *Leontopodium nanum*, and *Stipa purpurea*) was replaced by a monoculture of *Leontopodium nanum* within the ring (Figure 1a and Figure S1); (ii) T2 associated with *Agaricus campestris*, which exhibited a conspicuous belt of stimulated, luxuriant plant growth (Figure 1b and Figure S3); and T3 produced by *Clitocybe* sp., which showed no discernable shifts in plant composition, marked only by the circular emergence of sporocarps (Figure 1c and Figure S5). To capture the peak of fungal biological activity, sampling was carried out during the fruiting season, from July to September 2020. To minimize the impact of short‐term environmental variability, all samples for each specific FR were collected on the same day. To complement the morphological diagnoses presented in Figures S1, S3, and S5, we performed phylogenetic reconstructions using internal transcribed spacer (ITS) regions. These molecular analyses allowed for a robust comparative assessment of the study species against their nearest phylogenetic neighbors and type strains (Figures S2, S4, and S6). Taxonomic assignments were validated through combined morpho‐molecular analyses (Supporting Information). Following identification, all fungal collections were processed via forced‐air drying and archived in the repository of the Key Laboratory of Adaptation and Evolution of Plateau Biota, Chinese Academy of Sciences.

Representative circular or arc‐shaped FRs were chosen to investigate the impacts of *Agrocybe* sp., *A. campestris* , and *Clitocybe* sp. on the soil‐root interface, we employed a hierarchical sampling design to ensure spatial independence of biological replicates. For each FR type, five spatially independent rings (separated by at least 50 m to avoid overlapping influence) were selected as biological replicates. Within each individual ring, soil‐root pairs were collected from three discrete positional zones: the ON zone (fruiting body area), and the IN and OUT zones (located 20 cm inward and outward from the ON area, respectively). In total, 45 soil‐root pairs were collected at 10 cm depth (3 FR types × 5 independent rings × 3 positional zones = 45 samples). Root samples were collected from the dominant living plant species in each respective zone to represent the active root‐associated fungal communities under the prevailing field conditions. Specifically, in the T1 rings, we collected vital roots of *L. nanum* ; whereas in T2 and T3, we collected vital roots of *K. humilis*. All collected roots were confirmed viable via morphological inspection (intact, firm, cream‐colored root tips) prior to surface sterilization (70% ethanol 30 s, 5% NaClO 5 min, sterile water rinses) for endophyte DNA extraction. We characterized soil properties, including pH, MC, C:N ratio, and total/available nutrients (TC, TN, TP, TK, NO_3_^−^, NH_4_^+^), following the analytical frameworks described by previous research (Xing et al. 2023). Furthermore, the Shannon‐Wiener Index was employed to quantify plant diversity based on species coverage across the FR transects.

### 2.2 DNA Extraction, PCR Amplification and Sequencing

Total genomic DNA was isolated from soil samples using the FastDNA SPIN Kit for Soil (MP Biomedicals, CA, USA) following the manufacturer's protocols. For fungal community analysis, the ITS1 region was amplified using the primer pair ITS1F (5′‐CTT GGT CAT TTA GAG GAA GTA A‐3′) (Gardes and Bruns 1993) and ITS2R (5′‐GCT TAT TGA TAT GC‐3′) (White 1990). The resulting PCR amplicons were purified with the EasyPure Quick Gel Extraction Kit (TransGen Biotech, Beijing, China) before library preparation using the TruSeq DNA Sample Prep Kit (Illumina, San Diego, CA, USA). High‐throughput sequencing was performed on the Illumina HiSeq PE250 platform by Novogene Co. Ltd. (Beijing, China). Due to the total DNA quality from the root system of one of the OUT zones in T2 failing to meet sequencing requirements, a total of 44 samples were audited in this study. The raw sequence data have been deposited in the NCBI GenBank database under accession numbers SAMN49511100‐SAMN49511143.

### 2.3 Fairy‐Ring Fungal Identification

To identify the FR‐forming fungi, genomic DNA was extracted from mature fruiting bodies using the CTAB method. The ITS region was amplified with the universal primers ITS4 and ITS5 (White 1990), and the resulting sequences were deposited in GenBank (PV867262–PV867276). Preliminary taxonomic assignments were performed via BLASTn searches against the NCBI database. For refined phylogenetic placement, multiple sequence alignments were executed using MAFFT v.7.526 (Rozewicki et al. 2019) under default parameters. Maximum likelihood (ML) trees were subsequently reconstructed using FastTree v.2.2 (Price et al. 2010), employing the JTT + CAT model with 1000 bootstrap replicates to evaluate node support. Due to the high sequence identity between T2 isolates and *Agaricus campestris*, its phylogeny was resolved using a dataset restricted to the genus *Agaricus*, referencing the taxonomic framework of Zhao et al. (2020). In contrast, T1 and T3 isolates exhibited lower similarity to extant database entries, showing closest affinities to *Agrocybe* and *Clitocybe*, respectively. Consequently, broader reference datasets from the families Strophariaceae (Nicolás Niveiro 2020) (prioritizing published type sequences) and Clitocybaceae (He et al. 2023) were incorporated to precisely delineate their phylogenetic positions.

### 2.4 Bioinformatics and Statistical Analyses

Microbial community analysis was conducted using the QIIME 2 (v.2023.x) pipeline (Bolyen et al. 2019). Raw sequences were initially processed with FLASH (Magoč and Salzberg 2011), during which reads with average quality scores < 20 or lengths < 200 bp were filtered. The resulting high‐quality sequences were denoised into amplicon sequence variants (ASVs) via the DADA2 pipeline (Callahan et al. 2016). Taxonomic assignment of ASVs was performed using the RDP Classifier against the UNITE database (v.9.0). Sequences not belonging to the fungal kingdom were excluded from subsequent analyses (Quast et al. 2012). Since the initial taxonomic annotation did not explicitly identify the three target FR fungi, a local BLAST database was constructed utilizing our previously obtained ITS full‐length sequences from the fruiting bodies. To visualize the distribution and enrichment of ASVs across the zones for each type, ternary plots were constructed using the ggtern package in R (Hamilton and Ferry 2018). The ASVs exhibiting the highest sequence identity and lowest *E*‐values were identified as the core FR fungi: ASV144 (*Agrocybe* sp.), ASV1146 ( *A. campestris* ), and ASV86 (*Clitocybe* sp.). These assignments were further cross‐validated by NCBI BLAST searches, yielding consistent results. To explore the ecological interactions between these FR‐forming ASVs and the broader fungal community, Spearman's rank correlations were calculated using the psych package (v.2.5.3) in R (Revelle 2018). To account for variation in sequencing depth across samples, we applied Total Sum Scaling (TSS) normalization, in which the ASV abundance of each sample was divided by its total sequence count to obtain relative abundances. Rather than rarefying, TSS preserves all valid sequencing reads while effectively eliminating library‐size differences. To validate that sequencing depth was sufficient for downstream analyses, we calculated Good's coverage for each sample as Good's coverage = (1‐*n*_1_/*N*) × 100, where *n*_1_ is the number of singletons (ASVs occurring only once) and N is the total number of sequences in the sample. All samples exhibited 100% coverage (Table S1), indicating the complete absence of singletons and confirming that sequencing depth was fully saturated across all libraries. This validation justifies the use of TSS normalization for subsequent diversity and network analyses. Random Forest (RF) regression was performed using the randomForest package in R to identify key endophytic taxa predicting the abundance of the target FR fungi (Hamilton and Ferry 2018). The model was constructed with 1000 trees (*n*tree = 1000) using relative abundance data. Predictor importance was ranked based on the percentage increase in Mean Squared Error (%IncMSE) and increase in Node Purity (IncNodePurity).

The significance of differences in alpha‐diversity indices and the relative abundance of microbial taxa across FR zones was assessed using the Kruskal‐Wallis test, followed by the Benjamini‐Hochberg false discovery rate (FDR) correction for multiple comparisons. These analyses were implemented in the STAMP (v.2.1.3) software (Parks et al. 2014). To visualize beta‐diversity patterns, non‐metric multidimensional scaling (NMDS) based on Bray–Curtis distance matrices was performed using the vegan package (Simpson et al. 2015). Furthermore, to pinpoint individual ASVs with significant differential abundance between specific zones, we utilized the DESeq2 package (Love et al. 2014). ASVs were identified as differentially abundant based on a |log2 fold change| > 1 and an adjusted *p*‐value (*p*adj) < 0.05. To evaluate the influence of environmental filtering, Mantel tests were performed to assess the correlations between microbial community dissimilarity (Bray–Curtis distance) and both soil physicochemical properties (Supporting Information) and plant diversity (Shannon–Wiener Index) (Simpson et al. 2015). The distribution of unique and shared taxa across FR zones was visualized via Venn diagrams using the EVenn platform (Yang et al. 2024). Microbial interactions were further explored through co‐occurrence network analysis implemented in the R package meconetcomp (v.0.1.0) (Liu et al. 2023).

The networks were constructed based on SparCC (Sparse Correlations for Compositional data) to mitigate compositional bias, with significant correlations defined by a SparCC coefficient > 0.5 and *p* < 0.05. Global network properties, including the total number of nodes and edges, average degree, average path distance, and centralization metrics (betweenness and degree centrality), were calculated to characterize the topological stability of the communities. To identify potential keystone species, nodes were categorized based on their topological roles within and between modules. Following the criteria of within‐module connectivity (*Z*_*i*_) and among‐module connectivity (*P*_*i*_), nodes were classified as: (i) module hubs (*Z*_*i*_ ≥ 2.5, *P*_*i*_ < 0.62), (ii) connectors (*Z*_*i*_ < 2.5, Pi ≥ 0.62), or (iii) network hubs (*Z*_*i*_ ≥ 2.5, P_i_ ≥ 0.62). These three categories collectively represent the keystone nodes that maintain the structural integrity of the fungal and bacterial interactions. Fungal functional guilds were assigned using the MicrobiotaProcess (v1.14.0) R package (Xu et al. 2023) based on the FUNGuild database. Only “Highly Probable” and “Probable” functional assignments were retained. ASVs were classified into distinct trophic modes and guilds for subsequent statistical comparisons.

## 3 Results

### 3.1 Identification of Fairy‐Ring Fungi and Sequencing Overview

Sequence analysis of the ITS region revealed that the T2‐type FR fungus shares the highest homology with *Agaricus campestris*, which is fully corroborated by both phylogenetic and morphological evaluations (Figures S3, S4). In contrast, the T1 and T3‐type isolates displayed relatively low ITS sequence similarities (~80%) to known database entries (Figures S2 and S6). Despite this low molecular homology, comprehensive morphological characterizations allowed for the taxonomic assignment of the T1 and T3‐type FR fungi to the genera *Agrocybe* (Figure S1) and *Clitocybe* (Figure S5), respectively.

After quality filtering and denoising, a total of 1,374,085 high‐quality sequences were retained. To assess whether sequencing depth was sufficient to capture the full fungal diversity across samples, we calculated Good's coverage for each sample. All samples exhibited 100% coverage (Supporting Information), confirming the complete absence of singletons and indicating that sequencing depth was fully saturated. Regarding sample‐level depth, the lowest average read counts were observed in the ON zones of T2 and T3, whereas the IN and ON zones of T1 exhibited the highest sequencing depth. Sequence clustering or denoising identified 2193 ASVs. The ASV richness peaked in the ON zones of T1 and T2, as well as the OUT zone of T3 (Supporting Information). Across all fungal representative (FR) types, the T1 and T3 groups were characterized by the highest richness of unique ASVs distributed throughout all zones. In stark contrast, the T2 group exhibited a markedly lower level of zone specificity; unique ASVs accounted for a mere 7% of the total profile in the ON zone, while the OUT zone yielded no unique ASVs (Figure S7).

### 3.2 Taxonomic Composition and Diversity of Root Endophytic Fungal Communities

**FIGURE 2:**
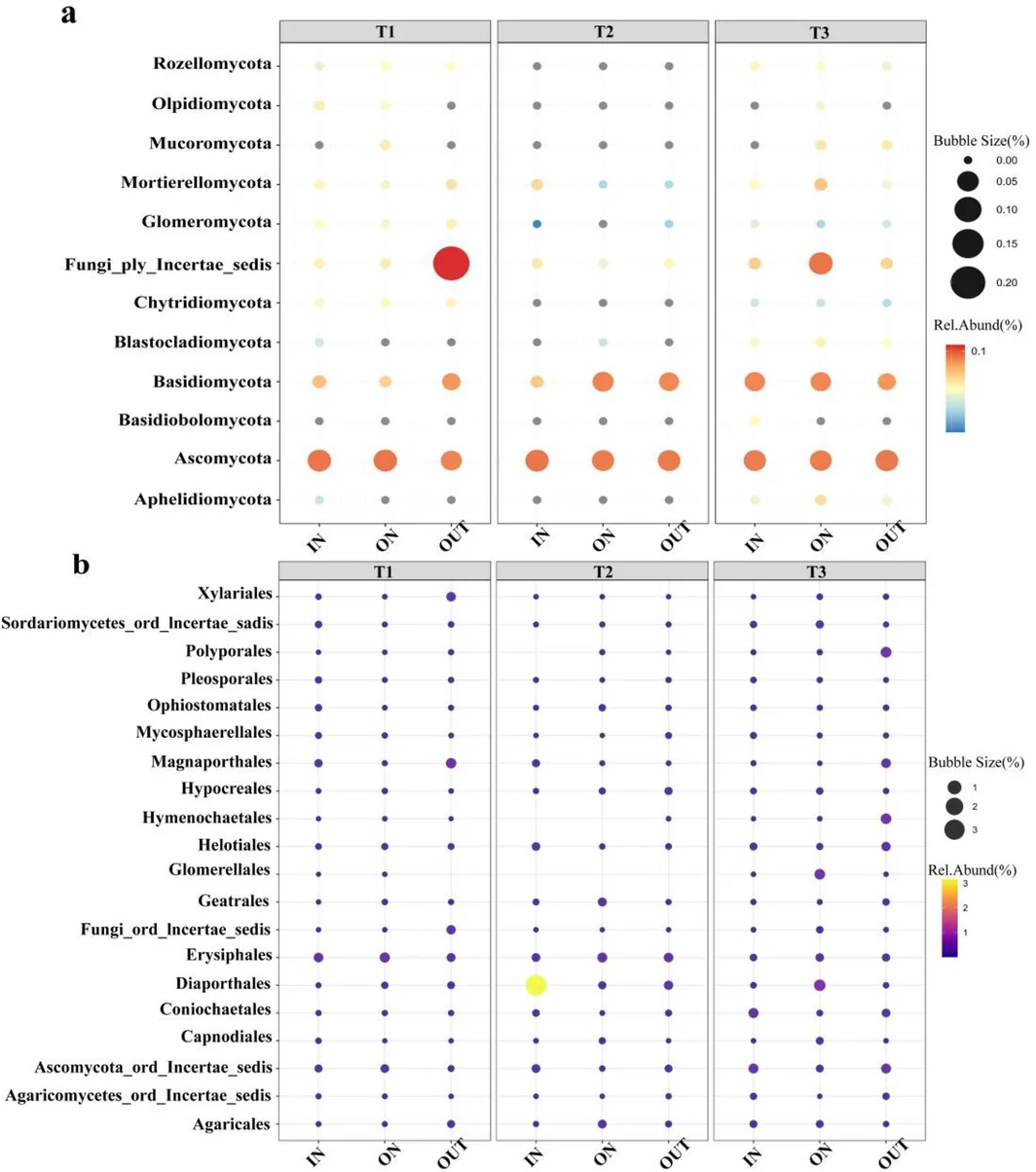
Taxonomic composition of root endophytic fungal communities across different fairy ring types and zones. Bubble plots showing the relative abundance of dominant fungal phyla (a) and classes (b) across the IN, ON, and OUT zones of T1, T2, and T3 fairy rings. The size and color intensity of the bubbles are proportional to the relative abundance of each taxon, with larger and darker bubbles indicating higher abundance.

Regarding the composition of endophytic fungal communities, the assemblages were primarily characterized by Ascomycota and Basidiomycota at the phylum level (Figure 2a), with the former exhibiting overall dominance. Further analysis at the class level revealed increased diversity, where Leotiomycetes, Sordariomycetes, Agaricomycetes, and Dothideomycetes emerged as the predominant taxa across all samples (Figure 2b). Notably, the relative abundance of Leotiomycetes was consistently higher in the ON zones. Specifically, Agaricomycetes prevailed in the T2 ON zone, while Dothideomycetes reached higher relative abundance in the T1 IN zone (Figure 2b). At the order level, community structures exhibited species‐driven divergence across FR types and zones; for instance, Diaporthales dominated the T2 IN zone, whereas Erysiphales predominated in the corresponding ON zone (Figure S8). Analysis of alpha and beta diversity across different types of FR revealed no significant differences between zones (Figure S9).

### 3.3 Differentially Abundant Endophytic Fungal Taxa Across Fairy‐Ring Zones

**FIGURE 3:**
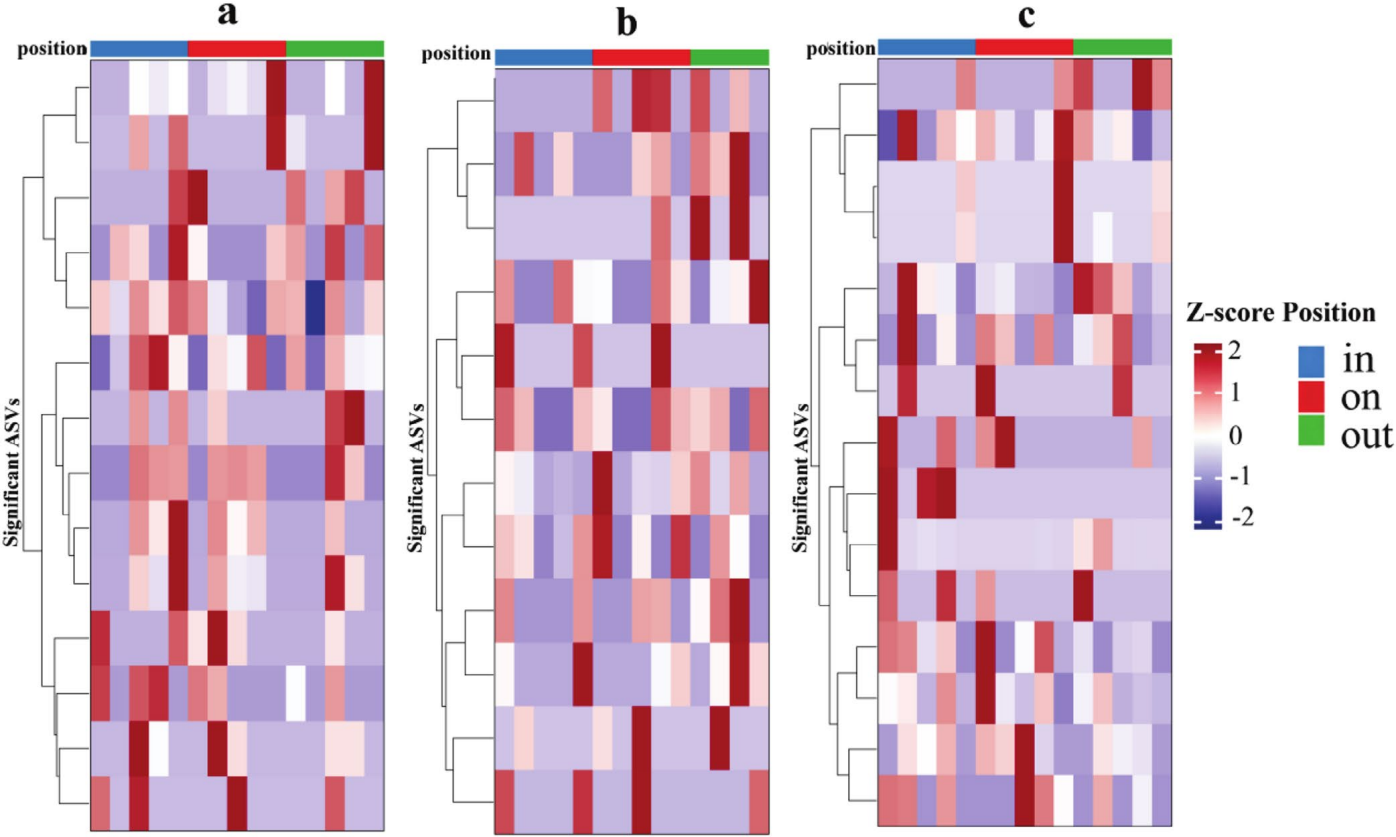
Hierarchical clustering heatmap of differentially abundant endophytic fungal ASVs across the IN, ON, and OUT zones of three fairy ring types. (a–c) Heatmaps for T1, T2, and T3 fairy rings, respectively. Rows represent individual ASVs identified as significantly differentially abundant by DESeq2 analysis (|log2FoldChange| > 1, *p*adj < 0.05). Columns represent individual samples from different zones. The color scale represents the *Z*‐score normalized relative abundance of each ASV: Red indicates high abundance (enrichment), while blue indicates low abundance (depletion). The dendrogram on the left shows the clustering of ASVs based on their abundance patterns across samples.

Based on the Kruskal‐Wallis test applied to the ternary plot distributions, only a limited number of ASVs were significantly enriched within their respective zones across the different fungal types (FRs) (Figure S10a–c). In contrast, a cross‐type comparison of the ON zones revealed a higher degree of taxonomic enrichment. Specifically, several fungal orders, including Pleosporales, Magnaporthales, Hypocreales, and Coniochaetales, exhibited significant enrichment within the ON zone of T1 (Figure S10d). DESeq2 analysis revealed that the T2 group exhibited the lowest number of significantly differential ASVs across all zones compared to other FR types. In T1, the IN zone was characterized by 798 significantly upregulated ASVs, which predominantly belonged to the classes Dothideomycetes, Leotiomycetes, Agaricomycetes, and Sordariomycetes (Figure 3a). For T2, the ON zone yielded 249 upregulated ASVs, primarily assigned to Leotiomycetes, Sordariomycetes, and Dothideomycetes. In contrast (Figure 3b), T3 demonstrated a relatively balanced distribution of upregulated and downregulated ASVs across all zones (Figure 3c). Notably, key symbiotic and root‐associated taxa, specifically within the Archaeorhizomycetes and Glomeromycetes, were found to be significantly downregulated in the ON zone across all FR types (Figure 3 and Supporting Information).

### 3.4 Environmental Drivers and Representative Fairy‐Ring Fungi

**FIGURE 4:**
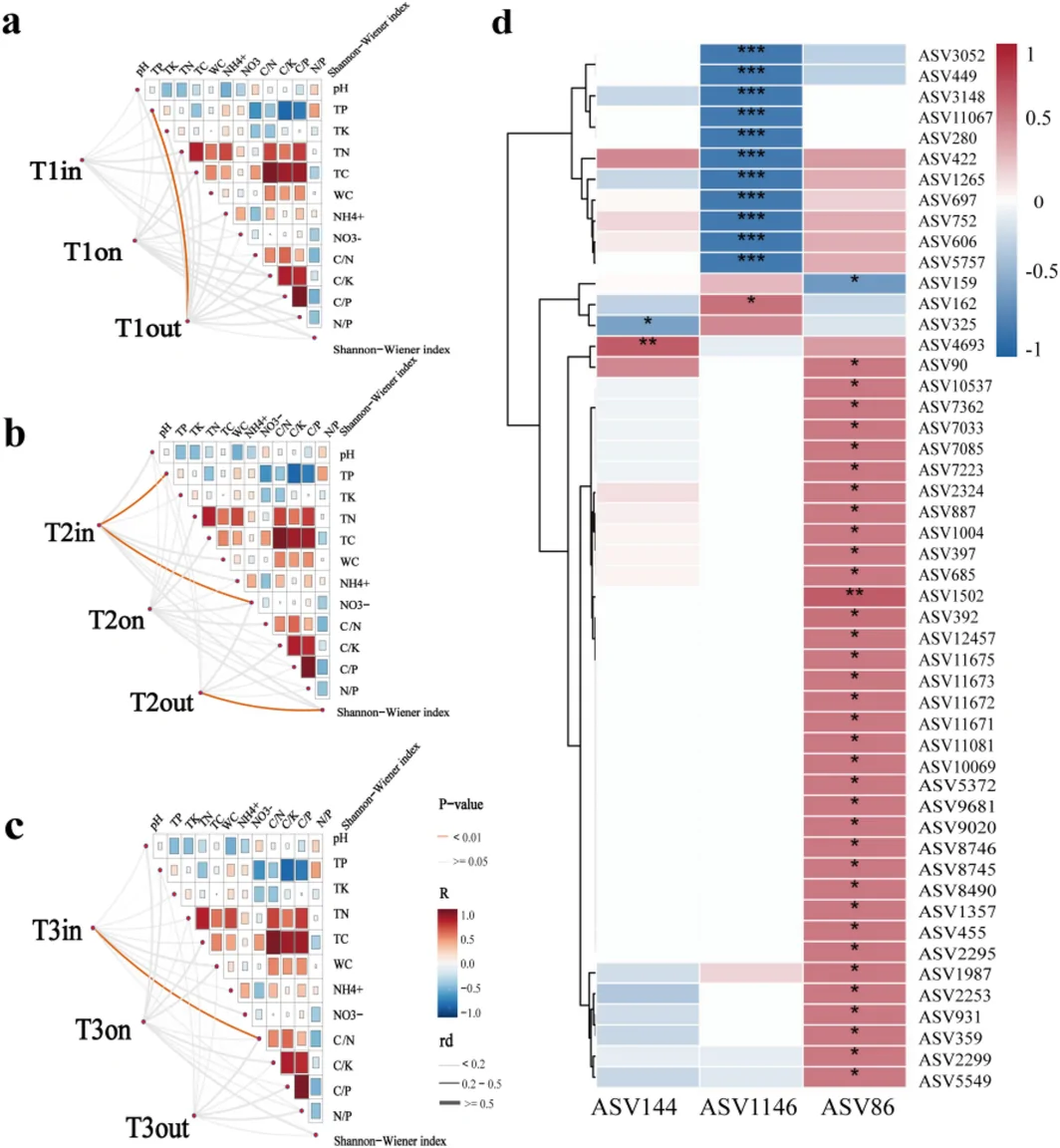
Environmental drivers of community variation and inter‐specific associations of fairy ring fungi. Mantel test analyses revealing the correlations between root endophytic fungal community structure and environmental variables across T1 (a), T2 (b), and T3 (c). The upper‐right triangular heatmap displays pairwise comparisons between environmental factors, with color gradients. Curved lines connect specific environmental variables to the community composition; line width is proportional to the Mantel's r statistic, and line color indicates statistical significance (e.g., orange lines indicate *p* < 0.05). Heatmap (d) illustrating the Pearson correlation analysis between the representative fairy ring forming fungi (ASV144 for T1, ASV1146 for T2, and ASV86 for T3) and other differentially abundant endophytic ASVs. MC, moisture content; TC, total carbon; TK, total potassium; TN, total nitrogen; TP, total phosphorus; WC, water content.

Mantel test analyses indicated that each zone was subject to significant regulation by a restricted set of environmental variables. Specifically, the endophytic fungal community in the T1 OUT zone was regulated only by TP (Figure 4a and Supporting Information), whereas in T2, the IN and OUT zones were driven by TP and nitrate nitrogen (Figure 4b and Supporting Information), and vegetation diversity, respectively. Following the criteria established in the Materials and Methods, ASV144, ASV1146, and ASV86 were identified as the representative FR fungi for the T1, T2, and T3 stages, respectively. Pearson correlation analysis revealed distinct inter‐specific interaction patterns: in T1, nine ASVs showed significant, predominantly negative correlations with ASV144, including members of the Mortierellomycetes, Agaricomycetes, Dothideomycetes, and Sordariomycetes (Figure 4d and Supporting Information). Similarly, T2 harbored 11 ASVs that were all negatively correlated with ASV1146, primarily belonging to Eurotiomycetes, Leotiomycetes, and Sordariomycetes (Figure 4d and Supporting Information). In contrast, the T3 stage exhibited a much broader and predominantly positive correlation network, with 54 ASVs significantly associated with ASV86, most notably from the Agaricostilbomycetes, Dothideomycetes, and Sordariomycetes (Figure 4d and Supporting Information). RF modeling was also employed to identify the key predictive ASVs contributing to the community dynamics across the three FR types (T1, T2, and T3). For the T1 stage (Target ASV144), the model identified ASV4693 (Chaetothyriales) as the most influential predictor (Importance: 0.226), exhibiting a strong positive correlation (*r* = 0.81) with the target fungus (Figure S11a). The top 20 predictive taxa in T1 were predominantly composed of Sordariomycetes (35%) and Leotiomycetes (25%), with a high proportion (85%) showing synergistic enrichment (positive correlation) with ASV144 (Figure S11a). In the T2 stage, ASV29 (Hypocreales) emerged as the primary contributor to model accuracy (Importance: 0.215), showing a robust positive association (*r* = 0.98) with the representative fungus (Figure S11b). The predictive community in T3 was characterized by the presence of Sordariomycetes, Leotiomycetes, and Ascomycota_cls_Incertae_sedis, with a more balanced mix of positive and negative responders (Figure S11c).

### 3.5 Co‐Occurrence Network Characteristics of Endophytic Fungal Communities

**FIGURE 5:**
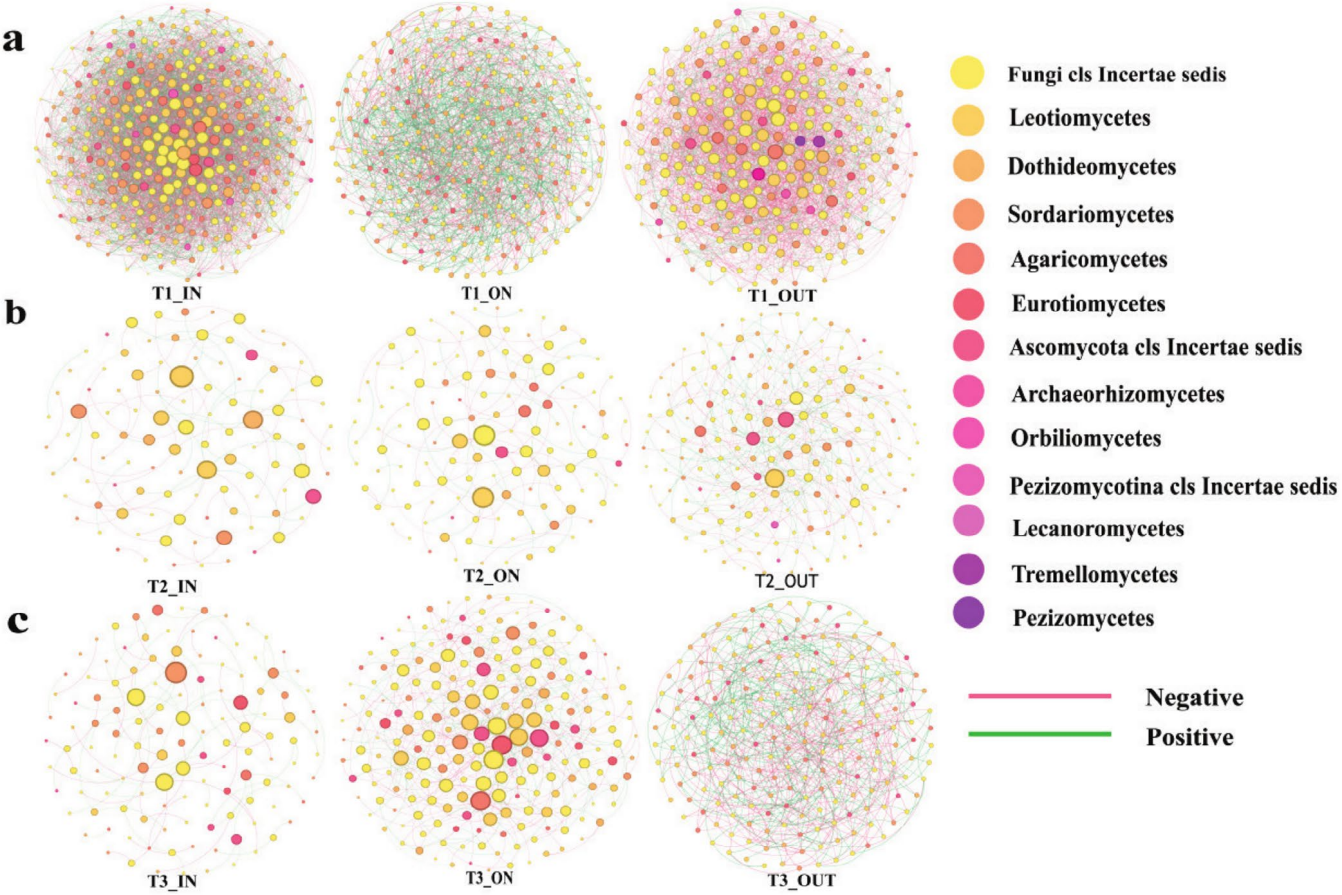
Intra‐kingdom co‐occurrence networks. Node size indicates the degree of connection. Edge color represents positive and negative correlations. a, b, and c are the co‐occurrence networks of the endophytic fungal communities of the T1, T2, and T3 types of fairy rings, respectively.

To elucidate the ecological interactions among endophytic fungi, co‐occurrence networks were constructed for each zone across the three FRs (T1, T2, and T3). The topological properties of these networks exhibited distinct spatial variations (Figure 5 and Table S1). All constructed networks possessed modularity indices greater than 0.4. In the T1, the network in the IN zone was the most complex, characterized by the highest number of nodes (89) and edges (165), as well as a relatively high average degree (3.71). In contrast, the ON zone network was smaller (58 nodes, 77 edges) but exhibited a higher modularity (0.60) compared to the IN zone (0.47). The OUT zone showed moderate complexity with an average degree of 4.65 but lower modularity (0.43) (Figure 5a and Supporting Information). For the T2 FR, the IN zone network, despite having the fewest nodes (31), displayed the highest clustering coefficient (0.16) and graph density (0.14) among all constructed networks. Conversely, the T2 OUT zone comprised a larger network (56 nodes) with moderate connectivity (average degree 3.18). Similar to T1, the T2 ON zone maintained a high modularity (0.61) (Figure 5b and Supporting Information). The T3 FR displayed the most pronounced intra‐ring variation. The T3 IN network was robust and complex, with a high average degree (4.57) and 144 edges. However, the network structure in the T3 OUT zone was remarkably simplified, featuring the lowest number of edges (34) and average degree (1.74), alongside the highest modularity (0.71) observed in this study (Figure 5c and Supporting Information). The analysis of node roles (Table S2) revealed that the majority of ASVs were classified as peripherals, indicating that most species had few links outside their own modules. A specific focus on the ON zones revealed a consistent trend of high compartmentalization. Across all three rings, the ON zones generally harbored fewer connectors (4 in T1‐ON, 5 in T2‐ON, and 4 in T3‐ON) compared to their respective IN or OUT zones (e.g., 20 in T1‐IN and 12 in T2‐OUT). However, a unique topological feature was identified in the T3‐ON zone. While no network hubs were detected in any zone, T3‐ON was the sole network to contain a module hub (1 node).

### 3.6 Predicted Trophic Guild Composition

**FIGURE 6:**
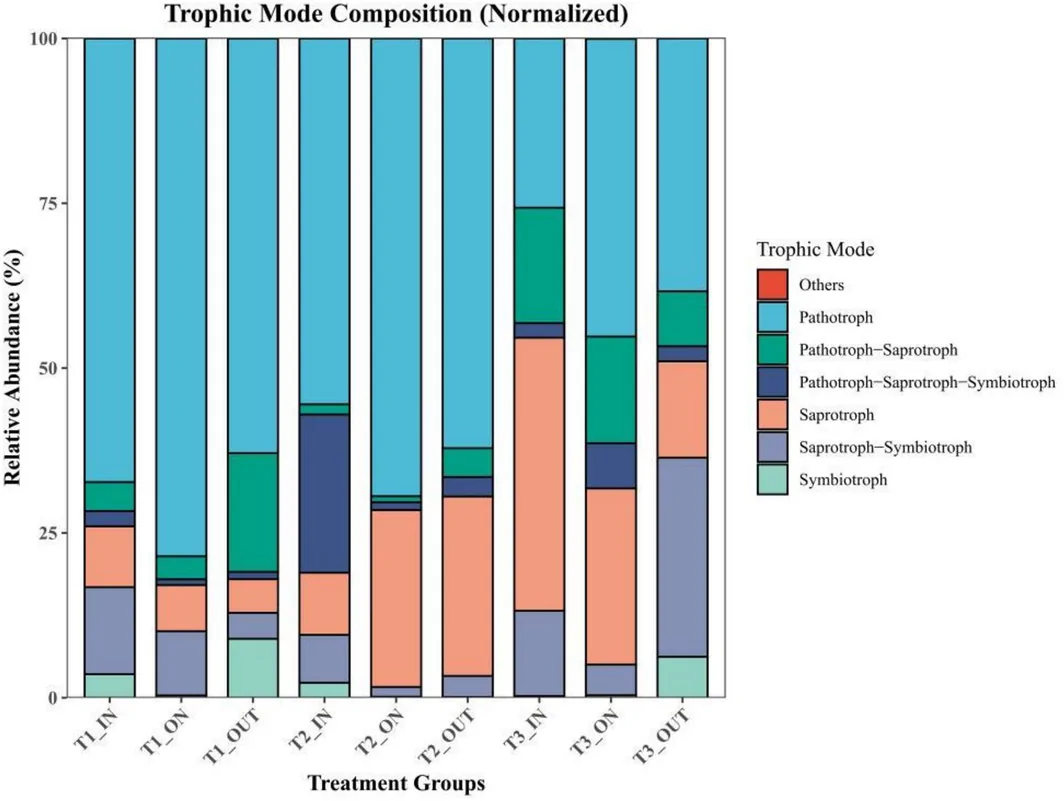
Predicted functional trophic modes of root endophytic fungal communities across different fairy ring types and zones.

The functional trophic modes of the endophytic fungal communities were predicted using FUNGuild to investigate their responses to different zones (IN, ON, and OUT) across three distinct FR types (T1, T2, and T3) (Figure 6). The predicted trophic guild composition exhibited significant spatial heterogeneity driven by the FR zones. In the T1 and T2 FRs, Pathotrophs were generally the dominant trophic mode across most zones, particularly in the ON zone, where they accounted for approximately 35%–39% of the total relative abundance (Figure 6). In contrast, the T3 FR showed a higher relative abundance of taxa assigned to Saprotrophs (Figure 6). The relative abundance of taxa assigned to Symbiotrophs was consistently suppressed in the ON zone across all FR types, a trend most notably exemplified by the T2 rings, where their proportion dropped to a minimum of 2.82%, significantly lower than the 33.54% and 6.26% observed in the inner (IN) and outer (OUT) zones, respectively (Figure 6).

## 4 Discussion

As “engineers” of soil ecosystems, FR fungi profoundly alter the rhizosphere microenvironment. By shaping the soil's physicochemical properties—acting as a “filter”—they selectively regulate and recruit microbial communities. First, fungi transform the pool of resources available to microbes by creating unique nutrient hotspots. Active fungal hyphae accelerate soil organic matter mineralization by secreting extracellular enzymes, leading to significant ammonium nitrogen (NH_4_^+^‐N) and readily available phosphorus (e.g., available phosphorus) enrichment at the hyphal front while simultaneously reducing total soil carbon and nitrogen pools (Caesar‐Tonthat et al. 2013; Du et al. 2024). Second, fungi induce physicochemical alterations in soil properties that directly screen microbial community composition. Within ON zones, fungi release hydrophobic proteins that markedly increase soil hydrophobicity (Zotti et al. 2021), while simultaneously lowering soil pH (Yang et al. 2018). These changes collectively constitute an “environmental filter” pressure on soil microbes. For example, in the FR ON zone of *Agaricus lilaceps*, the hydrophobic, nutrient‐rich rhizosphere microenvironment selectively enriched Gram‐negative, r‐strategy bacteria such as Pseudomonas (Caesar‐Tonthat et al. 2013). However, research on how FR affects REF communities remains extremely limited. The composition of plant endophytic bacterial communities follows the amplification‐selection model, which posits that microorganisms are sequentially selected in bulk soil, rhizosphere soil, and root tissues to form plant‐specific microbial communities (Wang et al. 2020). Consequently, root‐associated microbial communities demonstrate greater stability than rhizosphere communities when subjected to fungal disturbance by FR (Duan et al. 2022). For endophytic fungi, our investigation suggests that the assembly rules governing root endophytic fungal communities diverge fundamentally from this bacterial model. Unlike the bacterial microbiome, REF share a close evolutionary kinship and overlapping heterotrophic niches with the expanding FR fungi, as both belong to the fungal kingdom. As FR mycelia proliferate, they impose intense “intra‐kingdom competition” for colonization sites and carbon resources within the root endosphere (Rillig et al. 2025). This fundamental biological difference implies that the host's immune filtering or recruitment mechanisms—while effective for bacteria—may be insufficient to protect the root fungal microbiome from the invasive pressure of dominant FR fungi (Trivedi et al. 2020). Consequently, we observed a pattern of community reconfiguration, which we conceptualize as “symbiotic decoupling.” This term is used here as a working hypothesis to describe the reduced representation of certain root‐associated fungi in active FR zones. This pattern is reflected in the lower relative abundance of putatively symbiotic taxa, including Glomeromycetes (arbuscular mycorrhizal fungi) and Archaeorhizomycetes, within fungal‐active zones. The observed pattern may be consistent with competitive interactions among fungal taxa, potentially involving priority effects or metabolite‐mediated interactions, although this remains to be tested experimentally (Fukami 2015; Xing et al. 2022). These findings suggest that under FR perturbation, plants are forced to trade off the maintenance of costly symbiotic associations for the direct acquisition of FR‐mineralized nutrients (Johnson 2010). Thus, unlike bacterial communities, the composition of REF may be more strongly influenced by interactions among fungi themselves, highlighting the potential importance of intra‐kingdom interactions in shaping community patterns under FR expansion.

A prominent pattern associated with FR expansion in this study was a reduction in the representation of putatively symbiotic fungal taxa within active FR zones, a phenomenon that we conceptualize as “Symbiotic Decoupling.” Our data explicitly demonstrate a significant downregulation in the relative abundance of Archaeorhizomycetes and Glomeromycetes (arbuscular mycorrhizal fungi, AMF) within the ON zones across all FR types. This suppression was particularly pronounced in the growth‐promoting T2 (*Agaricus campestris*) rings: functional prediction analysis revealed that the relative abundance of symbiotrophs plummeted to a minimum of 2.82% in the ON zone, standing in sharp contrast to the levels observed in the inner (33.54%) and outer (6.26%) zones. These observations suggest a substantial reorganization of root‐associated fungal communities under FR influence. Although the mechanisms underlying this pattern cannot be directly resolved from the present dataset, the observed shifts are consistent with several ecological hypotheses. One possible explanation is a nutrient‐mediated trade‐off. Previous studies have shown that FR fungi can substantially increase the availability of phosphorus and other nutrients through accelerated organic matter mineralization (Kiers et al. 2011; Fisher 1977; Wang, Liu, et al. 2022). In our study system, total phosphorus was significantly enriched in ON zones and was identified as an important environmental factor associated with community assembly. Under such nutrient‐enriched conditions, the ecological importance of AMF‐mediated nutrient acquisition may be reduced, potentially decreasing the relative advantage of maintaining extensive symbiotic associations (Konvalinková et al. 2017; Raven et al. 2018). Consequently, the reduced abundance of AMF observed in ON zones may be consistent with a shift in plant–fungal interaction strategies under elevated nutrient availability(Konvalinková et al. 2017). A second, non‐exclusive explanation involves intensified interactions among fungal taxa. Because native endophytic fungi and FR fungi occupy overlapping functional niches within the fungal kingdom, increased competition for space and resources may occur during FR expansion (van der Wal et al. 2013). Several FR‐forming fungi, including *Marasmius oreades* and *Calocybe gambosa*, are known to produce a variety of secondary metabolites and volatile compounds that can influence surrounding microbial communities (Blenis et al. 2004; Zotti et al. 2021). Furthermore, the biomass dominance and high metabolic activity of FR fungi likely exert a “Priority Effect,” physically preempting colonization sites and blocking the re‐infection of AMF hyphae (Kennedy et al. 2009). Together, these processes may contribute to the reduced representation of symbiotic fungi observed in active FR zones, although direct evidence for these mechanisms remains lacking and will require targeted experimental validation.

Microbial co‐occurrence networks provide insights into patterns of microbial associations under different environmental conditions, although correlation‐based networks cannot directly infer ecological interactions or strategies. Our analysis indicates that root endophytic fungal communities exhibit contrasting network topologies across different FR types, suggesting divergent patterns of community organization under varying FR conditions. In the growth‐promoting T2 rings (*Agaricus campestris*), the network in the active ON zone exhibited a topology characterized by high modularity (0.61) but exhibited lower observed connectivity. As revealed in previous studies, the T2 ON zone is an environment of resource surplus, particularly enriched in bioavailable phosphorus (TP). Ecological theory suggests that in resource‐rich environments, the necessity for complex, facilitative microbial cooperation diminishes (Banerjee et al. 2018). Consequently, the endophytic network exhibits a pattern of relatively simplified network organization, which we conceptualize as ‘homogenization,’ with distinct modules dominated by taxa that may rapidly exploit nutrient pulses. This modular pattern may be consistent with niche partitioning among taxa within the root endosphere (de Vries et al. 2018). The observed modular topology may reflect a community pattern influenced by the presence of FR fungi and associated saprotrophs, rather than by intricate mutualistic cross‐feeding networks. Conversely, T1 rings exhibited a more densely connected network, a pattern we term ‘Defensive Complexity’. Such hyperconnected topology may be consistent with increased potential for community stabilization under stressful conditions, as suggested by previous studies of soil microbiomes (de Vries et al. 2018). Therefore, the hyperconnected topology observed in the T1 network may be consistent with patterns previously associated with microbial communities under environmental stress, although correlation‐based networks alone cannot determine whether this topology reflects enhanced stability or resistance. The core FR fungi (e.g., ASV1146 in T2) were strongly negatively correlated with numerous native endophytes. This pattern is consistent with intensified antagonistic associations within the fungal community, which may contribute to the observed differences in network structure between T1 and T2 rings (Faust and Raes 2012). It should be noted that the co‐occurrence networks presented here were inferred from correlation‐based associations, which provide insights into patterns of community organization but do not directly demonstrate ecological interactions or causal relationships. Therefore, the ecological interpretations proposed in this study should be regarded as plausible hypotheses that are consistent with the observed network patterns and existing ecological theory, while requiring further validation using complementary experimental or functional approaches.

The observed structural reorganization of the root endosphere was accompanied by notable shifts in predicted functional composition. FUNGuild analysis indicated that the relative abundance of Symbiotrophs in ON zones of T1 and T2 rings decreased, coinciding with an increase in Pathotrophs, which comprised approximately 35%–39% of total sequences. This pattern may reflect a functional shift from communities dominated by putatively mutualistic taxa toward those with greater representation of potential pathogenic or saprotrophic taxa, although the underlying mechanisms cannot be directly inferred from our data. In the injurious T1 FR, the increased representation of pathotrophic taxa is consistent with the hypothesis that environmental stress associated with FR fungi may influence endophytic community composition (“Secondary Infection” hypothesis). A previous study demonstrated that the mycelia of *Calocybe gambos* (Zotti et al. 2021), induce severe soil hydrophobicity, which has been associated with localized drought stress. Physical stress is known to compromise plant immune signaling (e.g., abscisic acid–salicylic acid antagonism), which could potentially affect root susceptibility to opportunistic colonization (Saijo and Loo 2020). Consequently, the relative increase of opportunistic taxa, including members of Dothideomycetes observed in our dataset, may reflect changes in endophytic community composition under these environmental conditions. While plant mortality has been observed in T1 rings, our data do not allow determination of the causal factors; the observed community shifts may indicate a pattern of dysbiosis associated with environmental stress. Even in the lush, growth‐promoting T2 FR, the enrichment of pathotrophs suggests a hidden ecological cost. This pattern is consistent with the “Growth‐Defense Trade‐off” hypothesis, which suggests that resource allocation to rapid growth may influence host defense investment (Huot et al. 2014). The high‐turnover root system in T2 may be associated with increased relative abundances of facultative saprotrophs and potential pathogens, suggesting that even nutrient‐favorable conditions can correspond with shifts in functional guild composition. In neutral T3 rings, the relative proportion of saprotrophs remained moderate and the network exhibited more positive interaction edges, indicating a relatively stable functional configuration in the absence of strong FR‐induced perturbation. The comparatively moderate representation of potential pathogens in T3 highlights the contrast in community composition and functional patterns between minimally perturbed and FR‐affected zones, without implying direct causation.

## 5 Conclusion

In summary, this study provides a comparative insight into how root endophytic fungal communities respond to the dynamic expansion of FRs. Our results highlight a pattern conceptualized as “Symbiotic Decoupling,” characterized by a reduction in the relative abundance of putatively mutualistic fungi, including AMF, in zones of high FR activity. The contrasting network topologies observed—simplified modules in nutrient‐rich Type II rings and more densely connected networks in Type I rings—may reflect different patterns of community organization under varying environmental contexts. We use the terms “Resource‐driven Intensification” and “Defensive Complexity” as conceptual descriptors for these observed patterns rather than confirmed strategies. While the observed patterns are consistent with hypotheses such as “Nutrient Trade‐off” and “Intra‐kingdom Competition,” we emphasize that these interpretations are based on community composition and functional predictions and do not constitute direct evidence for causal mechanisms. Future studies integrating transcriptomics, stable isotope tracing, and controlled inoculation experiments are needed to causally validate the metabolic exchanges between FR fungi, endophytes, and host plants. Nonetheless, our findings highlight the potential vulnerability of native symbiotic networks to fungal perturbations, suggesting that ecological restoration in alpine meadows should consider not only soil nutrients but also the resilience of the fungal microbiome. It should be noted that the trophic modes assigned by FUNGuild are inferred from taxonomic information rather than direct functional measurements. Therefore, the observed differences in predicted guild composition should be regarded as hypothesis‐generating patterns that warrant further validation using functional assays or multi‐omics approaches.

## Author Contributions

**Chengxing Wu:** conceptualization (equal), methodology (equal). **Zhihua Wu:** investigation (equal), methodology (equal). **Shiting Cao:** investigation (equal), software (equal). **Sisong Tu:** data curation (equal), investigation (equal). **Yuying Zhang:** data curation (equal), investigation (equal). **Na Li:** investigation (equal), methodology (equal). **Ming Wang:** investigation (equal), resources (equal). **Huiyuan Ren:** validation (equal). **Qingbo Gao:** formal analysis (equal), writing – review and editing (equal). **Shilong Chen:** formal analysis (equal), writing – review and editing (equal). **Rui Xing:** conceptualization (equal), data curation (equal), formal analysis (equal), funding acquisition (equal), investigation (equal), methodology (equal), project administration (equal), resources (equal), software (equal), supervision (equal), validation (equal), visualization (equal), writing – original draft (equal), writing – review and editing (equal).

## Funding

This work was supported by the Applied Basic Research Project of Qinghai Province (2023‐ZJ‐763); Qinghai Province's Kunlun Talents‐High‐end Innovative and Entrepreneurial Talents Program (QHKLYC‐GDCXCY‐2024‐120).

## Conflicts of Interest

The authors declare no conflicts of interest.

## Supporting information


**Figure S1:** Images of fruiting bodies and spores of the Type I fairy ring fungus *Agrocybe* sp.

**Figure S2:** A maximum likelihood phylogenetic tree of fungi containing Type I fairy ring fungi, including ITS sequences from fungi within the family Strophariaceae. Bootstrap values from 1000 replicates are shown as percentages at the branching points (Only values greater than 50 are displayed).
**Figure S3:** Images of fruiting bodies and spores of the Type II fairy ring fungus Agaricus campestris.
**Figure S4:** A maximum likelihood phylogenetic tree of fungi containing Type II fairy ring fungi, including ITS sequences from fungi within the genus Agaricus. Bootstrap values from 1000 replicates are shown as percentages at the branching points (Only values greater than 50 are displayed).
**Figure S5:** Images of fruiting bodies and spores of the Type III fairy ring fungus Clitocybe sp.
**Figure S6:** A maximum likelihood phylogenetic tree of fungi containing Type III fairy ring fungi, including ITS sequences from fungi within the family Clitocybaceae. Bootstrap values from 1000 replicates are shown as percentages at the branching points (Only values greater than 50 are displayed).
**Figure S7:** Venn plots show the distribution of endophytic fungal OTUs in different zones in the fairy rings (a: T1, b: T2, and c: T3).
**Figure S8:** Taxonomic composition of root endophytic fungal communities across different fairy ring types and zones. Bubble plots showing the relative abundance of dominant fungal order across the IN, On, and OUT zones of T1, T2, and T3 fairy rings. The size and color intensity of the bubbles are proportional to the relative abundance of each taxon, with larger and darker bubbles indicating higher abundance.
**Figure S9:** The α (a) and β (b) diversity of root endophytic fungal communities across different fairy ring types and zones.
**Figure S10:** Ternary plots depicting the distribution and enrichment patterns of root endophytic fungal ASVs across the IN, ON, and OUT zones of different FRs (T1:a, T2:b, and T3:c), and between ON zones of different FRs (d). Each point in the ternary plot represents an individual ASV, with its position determined by its relative abundance in the three respective zones. To identify significantly enriched taxa, a Kruskal–Wallis test was applied to each ASV (p < 0.05). Significant ASVs were color‐coded by their taxonomic Order.
**Figure S11:** Random Forest regression analysis identifying key endophytic predictors of fairy ring fungal abundance. (a–c) The top 20 most important predictor ASVs for the relative abundance of the core fairy ring fungi in TI (Agrocybe sp., Target ASV144), T2 (Agaricus campestris, Target ASV1146), and T3 (Clitocybe sp., Target ASV86) rings, respectively.
**Table S1:** Topology properties of the endophyticfungal intra‐kingdom networks.
**Table S2:** The distribution of keystone taxa in the endophytic fungal co‐occurrence network across different fairy rings and zones.

**Data S1:** Supplementary Methods.

**Data S2:** ece374301‐sup‐0004‐support_information.xlsx.

## Data Availability

The raw high‐throughput sequencing data generated in this study have been deposited in the NCBI GenBank database under accession numbers SAMN49511100‐SAMN49511143. The ITS sequences identifying the fairy ring‐forming fungi are available in GenBank under accession numbers PV867262–PV867276.
