# Supplementary material for: Structural and Functional Responses of Root Endophytic Fungi to Fairy Ring Expansion in Alpine Meadows on the Tibetan Plateau
Source: Ecol Evol. 2026 Sep 16;16(9):e74301. doi: 10.1002/ece3.74301 (PMC13581411; doi:10.1002/ece3.74301)
Supplement: Supplementary file 1 [file ECE3-16-e74301-s001.docx]

Fig.S1 Images of fruiting bodies and spores of the Type I fairy ring fungus *Agrocybe* sp.

Fig.S2 A Maximum Likelihood Phylogenetic tree of fungi containing type I fairy ring fungi, including ITS sequences from fungi within the family Strophariaceae. Bootstrap values from 1000 replicates are shown as percentages at the branching points (Only values greater than 50 are displayed).

Fig.S3 Images of fruiting bodies and spores of the Type II fairy ring fungus *Agaricus campestris*

Fig.S4 A Maximum Likelihood Phylogenetic tree of fungi containing type II fairy ring fungi, including ITS sequences from fungi within the genus *Agaricus*. Bootstrap values from 1000 replicates are shown as percentages at the branching points (Only values greater than 50 are displayed).

Fig.S5 Images of fruiting bodies and spores of the Type III fairy ring fungus *Clitocybe sp*.

Fig.S6 A Maximum Likelihood Phylogenetic tree of fungi containing type III fairy ring fungi, including ITS sequences from fungi within the family Clitocybaceae. Bootstrap values from 1000 replicates are shown as percentages at the branching points (Only values greater than 50 are displayed).

Fig. S7 Venn plots show the distribution of endophytic fungal OTUs in different zones in the fairy rings (a: T1, b: T2, and c: T3).

Fig. S8 Taxonomic composition of root endophytic fungal communities across different fairy ring types and zones. Bubble plots showing the relative abundance of dominant fungal order across the IN, On, and OUT zones of T1, T2, and T3 fairy rings. The size and color intensity of the bubbles are proportional to the relative abundance of each taxon, with larger and darker bubbles indicating higher abundance.

Fig. S9 The α (a)and β (b) diversity of root endophytic fungal communities across different fairy ring types and zones.

Fig. S10 Ternary plots depicting the distribution and enrichment patterns of root endophytic fungal ASVs across the IN, ON, and OUT zones of different FRs (T1:a, T2:b, and T3:c), and between ON zones of different FRs (d). Each point in the ternary plot represents an individual ASV, with its position determined by its relative abundance in the three respective zones. To identify significantly enriched taxa, a Kruskal-Wallis test was applied to each ASV (p < 0.05). Significant ASVs were color-coded by their taxonomic Order.

Fig. S11 Random Forest regression analysis identifying key endophytic predictors of fairy ring fungal abundance. (a–c) The top 20 most important predictor ASVs for the relative abundance of the core fairy ring fungi in TI (Agrocybe sp., Target ASV144), T2 (*Agaricus campestris*, Target ASV1146), and T3 (*Clitocybe* sp., Target ASV86) rings, respectively.

Table S1 Topology properties of the endophytic bacterial intra-kingdom networks.

| Networks | No. Node | No. edges | Average_degree | Average_path_length | Network_diameter | Clustering_coefficient | Density | Heterogeneity | Centralization | Modularity |
| --- | --- | --- | --- | --- | --- | --- | --- | --- | --- | --- |
| T1in | 89 | 165 | 3.71 | 3.03 | 7 | 0.05 | 0.04 | 0.59 | 0.07 | 0.47 |
| T1on | 58 | 77 | 2.66 | 2.98 | 6 | 0.04 | 0.05 | 0.63 | 0.09 | 0.60 |
| T1out | 68 | 158 | 4.65 | 2.39 | 5 | 0.12 | 0.07 | 0.53 | 0.14 | 0.43 |
| T2in | 31 | 66 | 4.26 | 2.26 | 5 | 0.16 | 0.14 | 0.40 | 0.09 | 0.41 |
| T2on | 41 | 49 | 2.39 | 3.27 | 7 | 0.03 | 0.06 | 0.57 | 0.07 | 0.61 |
| T2out | 56 | 89 | 3.18 | 2.92 | 7 | 0.07 | 0.06 | 0.47 | 0.05 | 0.50 |
| T3in | 63 | 144 | 4.57 | 2.28 | 5 | 0.07 | 0.07 | 0.51 | 0.09 | 0.40 |
| T3on | 88 | 114 | 2.59 | 3.77 | 9 | 0.00 | 0.03 | 0.63 | 0.10 | 0.66 |
| T3out | 39 | 34 | 1.74 | 4.05 | 9 | 0.00 | 0.05 | 0.54 | 0.06 | 0.71 |

Table S2 The distribution of keystone taxa in the endophytic bacterial co-occurrence network across different fairy rings and zones

| Networks | Network hubs | Module hubs | Peripheral nodes | Connectors |
| --- | --- | --- | --- | --- |
| T1_IN | — | — | 69 | 20 |
| T1_ON | — | — | 52 | 4 |
| T1_OUT | — | — | 54 | 11 |
| T2_IN | — | — | 26 | 5 |
| T2_ON | — | — | 34 | 5 |
| T2_OUT | — | — | 42 | 12 |
| T3_IN | — | — | 45 | 18 |
| T3_ON | — | 1 | 83 | 4 |
| T3_OUT | — | — | 32 | 1 |
|  |  |  |  |  |
