# Supplementary material for: Structural and Functional Responses of Root Endophytic Fungi to Fairy Ring Expansion in Alpine Meadows on the Tibetan Plateau
Source: Ecol Evol. 2026 Sep 16;16(9):e74301. doi: 10.1002/ece3.74301 (PMC13581411; doi:10.1002/ece3.74301)
Supplement: Supplementary file 2 [file ECE3-16-e74301-s002.docx]

“*Agrecybe* .sp_T1

mages of fruiting bodies and spores of the Type I fairy ring fungus *Agrocybe* sp.

Taxon Identification ReportIdentification: The specimen aligns with the generic circumscription of *Agrocybe* based on both morphological and micromorphological analyses:

1. Morphological Consistency: The specimen exhibits a glabrous (smooth) and non-viscid pileus, ranging from ochraceous to cream-white, which excludes the squamulose (scaly) genera such as *Pholiota*. The hygrophanous nature and the transition of lamellae color to a characteristic dull tobacco-brown (cigar-brown) at maturity are diagnostic markers for the family Bolbitiaceae, specifically the genus *Agrocybe*. The adnate attachment of the lamellae further supports this placement.

2. Micromorphological Analysis: The microscopic features are definitive for *Agrocybe*. Basidiospores: The spores are smooth, thick-walled (often double-walled), and pigmented yellowish-brown to tobacco-brown under light microscopy. Germ Pore: A distinct, prominent truncate germ pore is present at the apex, a critical taxonomic feature that distinguishes *Agrocybe* from allied genera with less developed pores. Spore Biometrics: The measured dimensions (6.5)9.1~13.6(14.0) × (4.9) 5.1~8.4(9.0) μm with a mean quotient (Q_m_ = 1.78) fall within the recorded range for several species within the *Agrocybe praecox* complex or related large-spored taxa.

3. Conclusion：Based on the integration of a glabrous, non-viscid pileus, tobacco-brown lamellae, and smooth, thick-walled basidiospores possessing a conspicuous truncate germ pore, the specimen is confidently identified as a member of the genus *Agrocybe*.

Differentiation from Allied Taxa within *Agrocybe* While the QTP Specimen shares core generic traits with *Agrocybe*—such as a truncate germ pore and tobacco-brown spores—it exhibits significant morphological and biometric divergence from the three most common congeners: *A. praecox, A. putaminum, and A. pediades*.

1. Distinction from *Agrocybe praecox* (Pers.) Fayod *A. praecox* is a relatively robust species (pileus 30~100 mm) characterized by a distinct, though sometimes evanescent, membranous annulus. Morphological Divergence: The QTP specimen is diminutive (pileus 5 mm) and entirely lacks any annular remnants. Biometric Divergence: The spores of *A. praecox* are significantly smaller, typically 8.0~11.0 ×5.0~7.0 μm. The QTP specimen’s spore length (up to14.0 μm) and breadth (up to 9.0 μm) markedly exceed the known range for the *A. praeco*x complex.

2. Distinction from *Agrocybe putaminum* (Maire) Singe *A. putaminum* is often confused with other *Agrocybe* species due to its spore size, but it is ecologically and texturally distinct. The pileus of *A. putaminum* is notably pruinose to velutinous (velvety) and often possesses a strong farinaceous odor. In contrast, the QTP specimen features a strictly glabrous (smooth) pileus surface. Ecology: *A. putaminum* is primarily a synanthropic species found on wood chips or garden mulch in temperate regions. The occurrence of the QTP specimen in pristine alpine meadows at high altitudes represents a fundamentally different ecological niche.

3. Distinction from *Agrocybe pediades* (Fr.) Fayod *A. pediades* is the most closely related taxon due to its preference for open grasslands and similar spore dimensions. However, it can be excluded based on spore geometry and habit. Spore Shape and Q Value: Although *A. pediades* can produce large spores (the so-called "large-spored" variants), they are typically more ovoid or broadly ellipsoid, with a lower mean Q value (typically 1.45~1.60). The QTP specimen exhibits a significantly higher Q_m_ = 1.78, indicating more elongated-ellipsoid spores. Extreme Diminutiveness: While *A. pediades* is a small species, its pileus typically measures 10~40 mm. The nanoid habit of our specimen (pileus 5 mm, height 7 mm) is exceptionally rare for *A. pediades* and suggests a distinct evolutionary adaptation to the extreme conditions of the Qinghai-Tibet Plateau.

*Agaricus campestris_*T2

 Images of fruiting bodies and spores of the Type II fairy ring fungus *Agaricus campestris*

Pileus: 8~12 cm diam, hemispherical or convex when young, expanding to applanate, sometimes slightly depressed at disk. Surface dry, white to greyish-white, initially silky, becoming appressed-fibrillose squamulose, often with pale brownish tones in age. Margin initially involute, often decorated with white veil remnants.

Lamellae: Free, crowded, unequal (lamellulae present). Color initially bright pink, becoming flesh-pink, finally dark chocolate-brown. Stipe: 3~8 cm long, 1~2 cm thick, cylindrical, sometimes tapering towards the base, central, solid. White, smooth above the annulus, occasionally sparsely fibrillose-squamulose below. Base context unchanging when cut. Context: White, thick, firm. Unchanging or slightly staining pinkish on exposure. Basidiospores: (6.5) 7.0~8.5 (9.0) × 4.5~5.5 (6.0) μm, Q_m_ =1.28, broadly ellipsoid to ovoid, smooth, inamyloid, lacking a germ pore.

In summary, combined with evidence from the phylogenetic tree, it can be concluded that this species is *Agaricus campestris*.

*Clitocybe.sp _*T3

 Images of fruiting bodies and spores of the Type III fairy ring fungus *Clitocybe sp*.

Taxonomic Description of the Specimen: Basidiomata medium-sized, characterized by a relatively short-stiped habit. Pileus 2.9~5.8 cm in diameter, initially plano-convex, becoming centrally depressed to typically infundibuliform at maturity; margin initially involute, later expanding and occasionally becoming wavy; surface glabrous, dry, ranging from white to cream or pale ochraceous. Lamellae deeply decurrent, moderately crowded to crowded, white to pale yellowish-cream. Stipe 1.6~2.2 cm in length, 0.5~1.2 cm thick, central, cylindrical or slightly tapering downwards, fibrous, internally solid to stuffed, concolorous with the pileus or slightly paler. Basidiospores (3.9) 4.2~7.3 (8.3)×(2.5) 2.8~6.2 (7.1) μm ( Q_m_ = 1.48); shape ellipsoid to broadly ellipsoid; hyaline, thin-walled, surface strictly glabrous under light microscopy, inamyloid.

Discussion: Differentiation from *Collybia tibetica* M. Zang, the specimen demonstrates clear morphological and biometric divergence from *Collybia tibetica*, a species primarily documented from the Qinghai-Tibet Plateau. The following diagnostic characters provide robust evidence against conspecificity:

1. Lamellae Attachment (Primary Diagnostic Marker): the most significant distinction lies in the hymenophore configuration. Our specimen possesses deeply decurrent lamellae, a hallmark of the genus *Clitocybe*. In contrast, *Collybia tibetica* is characterized by adnate to adnexed lamellae, which do not extend down the stipe. This fundamental difference in gill attachment indicates a different phylogenetic lineage within the Agaricales.

2. Pileus Morphology and Habit: the macromorphology of the current specimen is strictly infundibuliform (funnel-shaped) with a deeply depressed center at maturity and an involute margin. *C. tibetica*, according to the original description, typically exhibits a convex to plano-convex pileus that rarely becomes truly infundibuliform. Furthermore, our specimen displays a squat habit (pileus width 2.9~5.8 cm vs. stipe height 1.6~2.2cm ), whereas *C. tibetica* generally possesses a relatively longer, more slender stipe.

3. Spore Biometrics and Q_m_ Values: a comparison of the basidiospore data reveals significant differences: Current Specimen: (3.9)4.2~7.3 (8.3)×(2.5)2.8~6.2(7.1) μm, with a Q_m_ = 1.48. This indicates a predominantly ellipsoid spore shape. The spores of *C. tibetica* are typically smaller and narrower, often described with a different range. The breadth of our specimen's spores significantly exceeds the circumscription of C. tibetica. Ecology and distribution: Saprotrophic, solitary to gregarious. Currently known from the alpine meadows of the Qinghai-Tibet Plateau (QTP), typically occurring on humus-rich soil among alpine grasses at high altitudes.”
